# A Nonlinear Rate Microsensor utilising Internal Resonance

**DOI:** 10.1038/s41598-019-44669-3

**Published:** 2019-06-17

**Authors:** Atabak Sarrafan, Soheil Azimi, Farid Golnaraghi, Behraad Bahreyni

**Affiliations:** Simon Fraser University, Surrey, BC V3T 0A3 Canada

**Keywords:** Electrical and electronic engineering, Mechanical engineering

## Abstract

Micro- and nano-resonators have been studied extensively both for the scientific viewpoint to understand basic interactions at small scales as well as for applied research to build sensors and mechanical signal processors. Majority of the resonant microsystems, particularly those manufactured at a large scale, have employed simple mechanical structures with one dominant resonant mode, such as in timing resonators, or linearly coupled resonant modes, as in vibratory gyroscopes. There is an increasing interest in the development of models and methods to better understand the nonlinear interactions at micro- and nano-scales and also to potentially improve the performance of the existing devices in the market beyond limits permissible by the linear effects. Internal resonance is a phenomenon that allows for nonlinear coupling and energy transfer between different vibration modes of a properly designed system. Herein, for the first time, we describe and experimentally demonstrate the potential for employing internal resonance for detection of angular rate signals, where the Coriolis effect modifies the energy coupling between the distinct drive and sense vibration modes. In doing so, in addition to providing a robust method of exciting the desired mode, the proposed approach further alleviates the mode-matching requirements and reduces instabilities due to the cross-coupling between the modes in current linear vibratory gyroscopes.

## Introduction

Micro- and nano-fabricated resonators are used in numerous applications including timing and frequency references, filters, chemical sensing, and physical sensing. Small-amplitude dynamics of these systems are typically studied using well-established methods such as Euler-Bernoulli theory for oscillating beams^1^. Various phenomena such as large-amplitude operation^2^ and material nonlinearities^3^ can cause a device response to deviate from linear predictions. In typical microsystem applications, such nonlinear effects are often avoided. Over the past decade, there has been growing interest in developing design methodologies for microsystems that exhibit different types of nonlinearities for basic research^4–8^ and in employing nonlinearities to improve the performance of micro- and nano-resonant systems, for instance, to improve the sensitivity of sensors^9,10^ or the stability of timing references^11^. Internal resonance is a particular nonlinear phenomenon that can cause nonlinear modal interactions between vibration modes directly excited by external harmonic forces and other vibration modes. Internal resonance may occur when the linear natural frequencies of a system are commensurate or nearly commensurate (e.g., *ω*_2_ = *kω*_1_ or *ω*_2_ ≈ *kω*_1_ where *k* = 2, 3, …) and there exist nonlinear coupling terms between the vibration modes^12^. Internal resonance has been studied from different perspectives because of the interesting dynamic properties. Consider a two degree-of-freedom (DOF) system where the equations of motion are:

1 $$\{\begin{array}{l}\ddot{x}+{\gamma }_{1}\dot{x}+{\omega }_{1}^{2}x=f(x,y)+F\,\cos ({{\rm{\Omega }}}_{r}t+{{\rm{\Phi }}}_{1})\\ \ddot{y}+{\gamma }_{2}\,\dot{y}+{\omega }_{2}^{2}y=g(x,y)\end{array}$$

where the first equation represents the externally excited mode through input force $F\,\cos ({{\rm{\Omega }}}_{r}t+{{\rm{\Phi }}}_{1})$ and the second equation represents the indirectly excited sub-system. Parameters *γ*_1_ and *γ*_2_ are the damping coefficients while *ω*_1_ and *ω*_2_ are the natural frequencies of the undamped linear oscillators for the vibrational modes, respectively. Functions *f*(*x*, *y*) and *g*(*x*, *y*) represent the coupling between the two modes. In linear Coriolis vibratory gyroscopes (CVGs), for instance, these functions will be anti-symmetric (i.e., *f*(*x*, *y*) = −*g*(*x*, *y*) and $f(x,y)\propto m\dot{y}{\rm{\Omega }}$. Nonlinear 2:1 internal resonance occurs as a result of quadratic nonlinearities present in the system when Ω_*r*_ ≈ *ω*_1_, *ω*_1_ ≈ 2*ω*_2_, $f(x,y)={\dot{y}}^{2}$ and $g(x,y)=2\dot{x}\dot{y}$. In this case, the nonlinear quadratic coupling terms lead to auto-parametric excitation of the lower-frequency mode by the higher-frequency mode^13^. In other words, the vibration energy from the mode with a higher natural frequency is pumped into the mode of lower natural frequency. The amount of energy that is transferred depends on the type of quadratic nonlinearities, the amplitude of external force, modal Q-factors, and the frequency ratio between the vibrational modes, among others^8^. An interesting characteristic of internally resonant systems with 2:1 frequency ratio in the presence of quadratic coupling nonlinearities is *saturation*. When the system is excited at a frequency near the higher natural frequency, the structure responds to the frequency of excitation, and the amplitude of the response increases linearly with the amplitude of excitation^13^. However, when the vibrational amplitude of this mode reaches a threshold value, it saturates and the additional energy from the input source is transferred to the lower natural frequency mode due to the nonlinear coupling between them. The mode with the lower resonant frequency then starts to oscillate at half the excitation frequency and its amplitude grows proportional to the spill-over energy from the mode directly excited by the input. In macro-systems, internal resonance has been mainly studied to better understand the modal interactions with the intention of suppressing unwanted vibrations^14–16^. However, practical applications of internal resonance at micro- and nano-scales have remained limited to basic demonstrations of the phenomena^8,17^, taking advantage of the saturation phenomenon for amplitude and frequency stabilization^11^, and exploiting the phenomenon for mass-sensing^18^.

An application area that requires two coupled resonators and can potentially benefit from internal resonance is the measurement of angular rate. Gyroscopes made using microelectromechanical systems (MEMS) technologies are used increasingly in applications ranging from navigation and robotics to stability control due to their small size, low power consumption and low cost. Many MEMS gyroscopes operate based on Coriolis effect where the Coriolis acceleration couples two structural modes of vibration (i.e., sense and drive) dynamically. To maximize the sensitivity of MEMS Coriolis vibratory gyroscopes (CVGs), the natural frequencies of the sense and drive modes are designed to match. However, perfect matching between these modes is challenging because of the manufacturing nonidealities and tolerances. Moreover, maintaining the frequency matching during the operation of the gyroscope usually is not possible since parameter fluctuations under operating conditions may induce further mistuning. Proposed solutions for mode-matching include post-processing techniques^19^, active electronic control systems^20^, and structural design improvements^21^. Inherent robustness may be achieved by widening the bandwidth of the sense mode through linear coupling of vibration modes^22^. The amount of frequency split can be tuned using voltage signals^23^. Calculation of the required excitation signals can be carried through, for instance, statistical learning methods for the automatic mode-matching circuit and elimination of the frequency split^24^. It has also been proposed that by employing parametric resonance, the resonance frequency of the drive mode can be varied as needed until the sense mode is excited^25^.

In this work, we are proposing a fundamentally different approach to improve the robustness of a MEMS gyroscope response to design parameter variations. The proposed design exploits the 2:1 internal resonance within a microresonator with 2 DOF such that the sense mode bandwidth is widened to the extent to circumvent the requirement for mode matching. The phenomenon is self-initiating once the excitation amplitude exceeds a threshold level without a need for a controller, electrostatic tuning, or additional DOF.

## Results

### Device design

**Figure 1:**
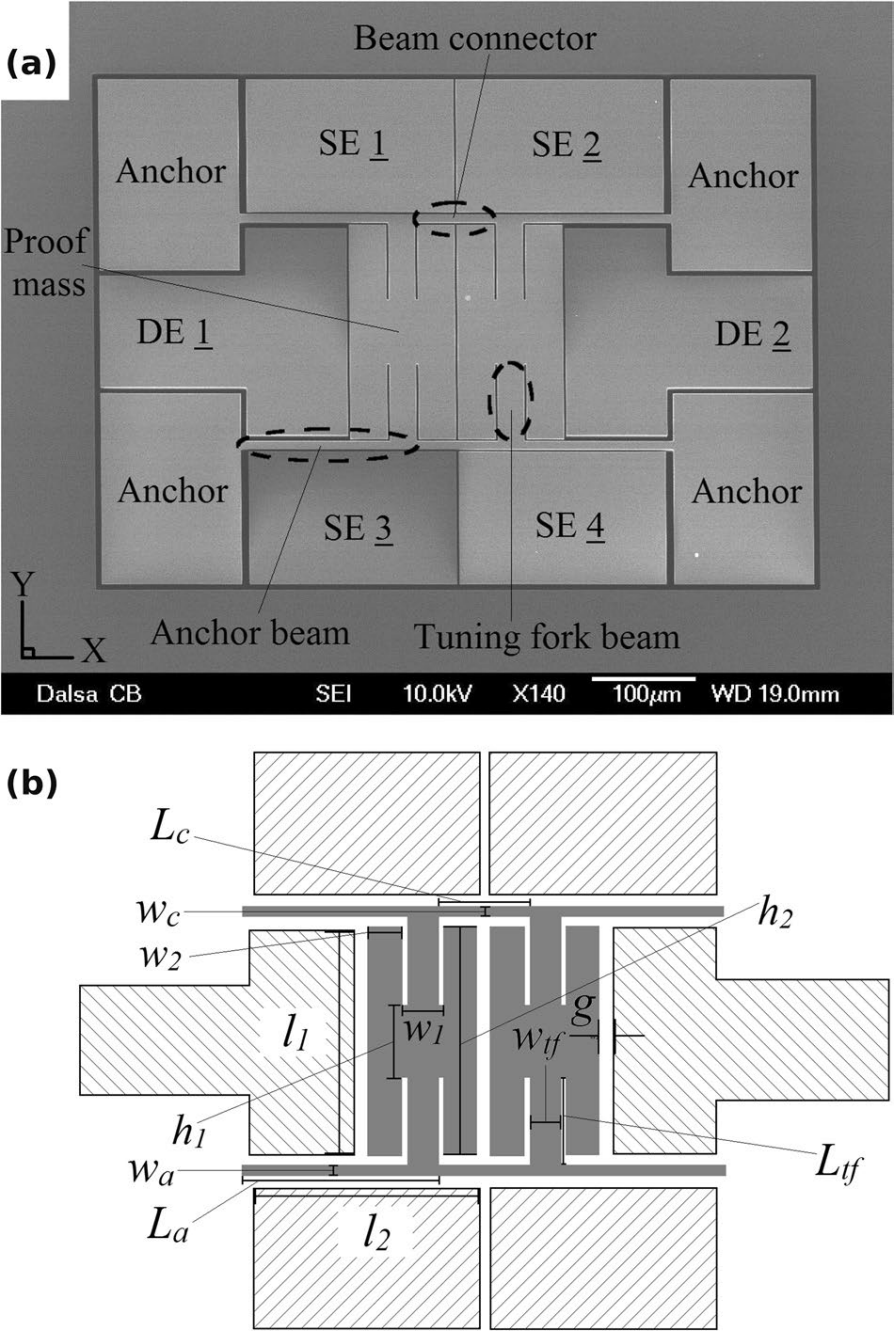
(**a**) Top-view SEM image of the resonator device before vacuum encapsulation. (**b**) Device dimensions and design parameters.

The device utilises twin proof masses that are mechanically connected to two crossbar suspensions forming a tuning fork type resonator as shown in Fig. 1. Slender flexural beams are used for the connections between the device components and to the substrate. The device is excited by applying input voltages to the two parallel plate drive electrodes (DE) 1 and 2 to exert an electrostatic force onto the structure. Movements of the structure are measured using a combination of capacitive sense currents from the sense electrodes (SE) 1–4. Under small forces, the device remains linear with two separate modes. With an adequately large driving force, the microresonator behaves a spring-pendulum mechanism with quadratic nonlinearities (see the Supplementary Information attachment on modeling of the system)^26^. Nonlinearities at small scales can stem from structural, electrostatic, and material origins. In this particular case, large displacements will result in quadratic nonlinearities due to the combined linear and angular movements of the structural components. The twin proof masses can move in the radial direction (spring mode) or rotate about the anchor (pendulum mode), simultaneously. Under these conditions, there can be a nonlinear coupling of vibration energy from the forced vibrations along the spring direction to the pendulum mode through 2:1 internal resonance because of the inherent quadratic nonlinearities in the system. Taking into account the quadratic Coriolis and centripetal nonlinearities, the system can be modeled using different methods, including the perturbation method (see the Supplementary Information attachment)^27^. The designed microdevice prioritizes anti-phase resonant modes such that the natural frequency of the anti-phase resonant mode along the *x*-axis (spring mode) is twice the frequency of the anti-phase resonant mode along the *y*-axis (pendulum mode). The fabricated device is vacuum sealed through a hermetic, wafer-level process before dicing.

### Linear response characterisation

**Figure 2:**
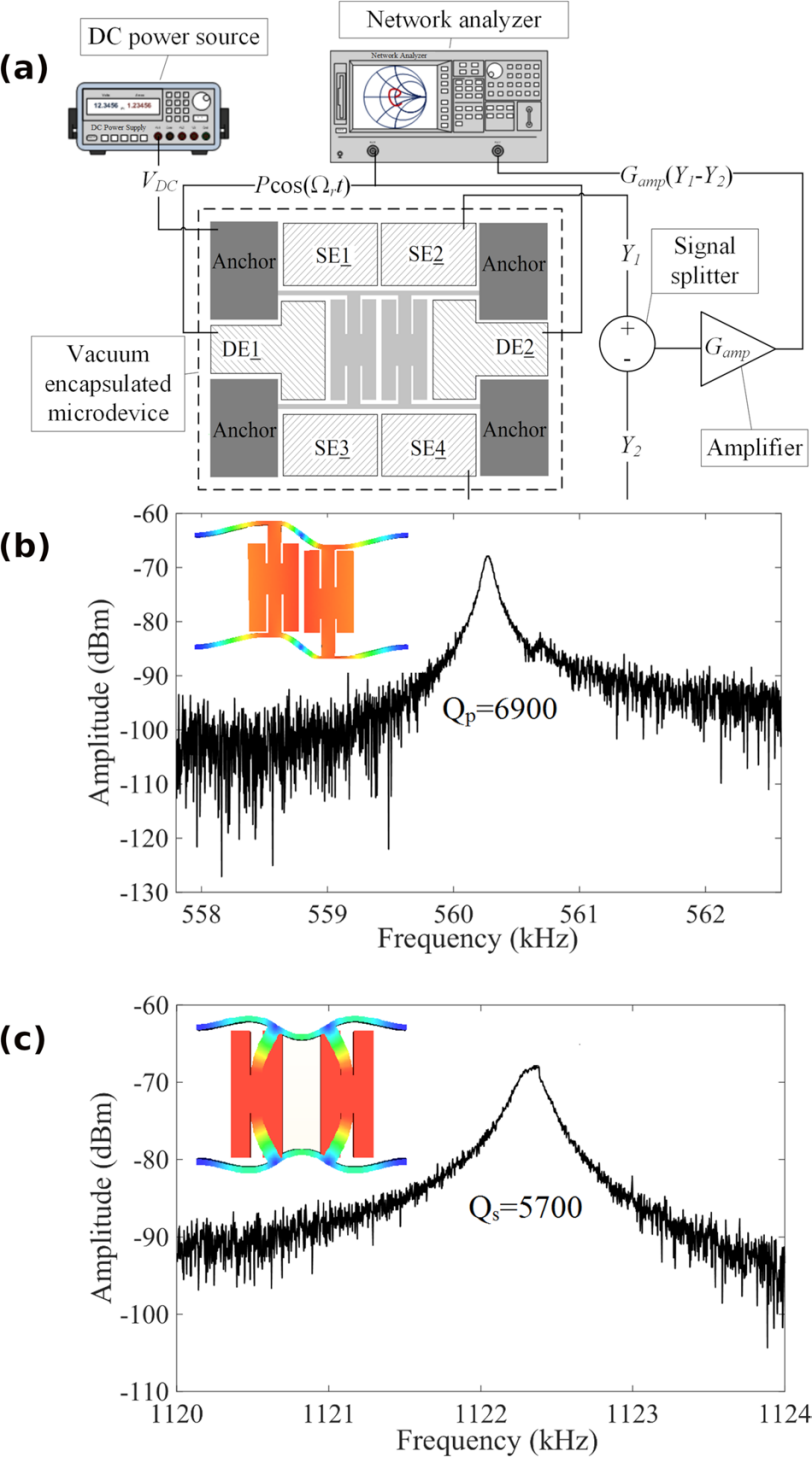
(**a**) The experimental configuration to measure the linear natural frequencies and the quality factors of the vibration modes. The measured frequency spectrum in (**b**,**c**) demonstrates the frequency response and quality factor of the pendulum and spring modes at the onset of nonlinearities, respectively.

The natural frequencies of the microresonator (*f*_*s*_ and *f*_*p*_) and the quality factors (*Q*_*s*_ and *Q*_*p*_) for the spring and pendulum modes are measured using the test-bed shown in Fig. 2a. A vector network analyzer was used to measure the device response at the frequency of the input signal. A DC bias voltage is used to enhance the linear component of the input electrostatic force. Measured spectra around the spring and pendulum mode frequencies at the onset of nonlinearities are shown in Fig. 2b,c, respectively. For the device studied here, *f*_*p*_ = 560.28 *kHz* with *Q*_*p*_ = 6900 (pendulum mode) and *f*_*s*_ = 1122.35 *kHz* with *Q*_*s*_ = 5700 (spring mode), leading to a frequency ratio between spring and pendulum modes of *f*_*s*_/*f*_*p*_ = 2.003 ≈ 2.

### 2:1 Internal resonance

**Figure 3:**
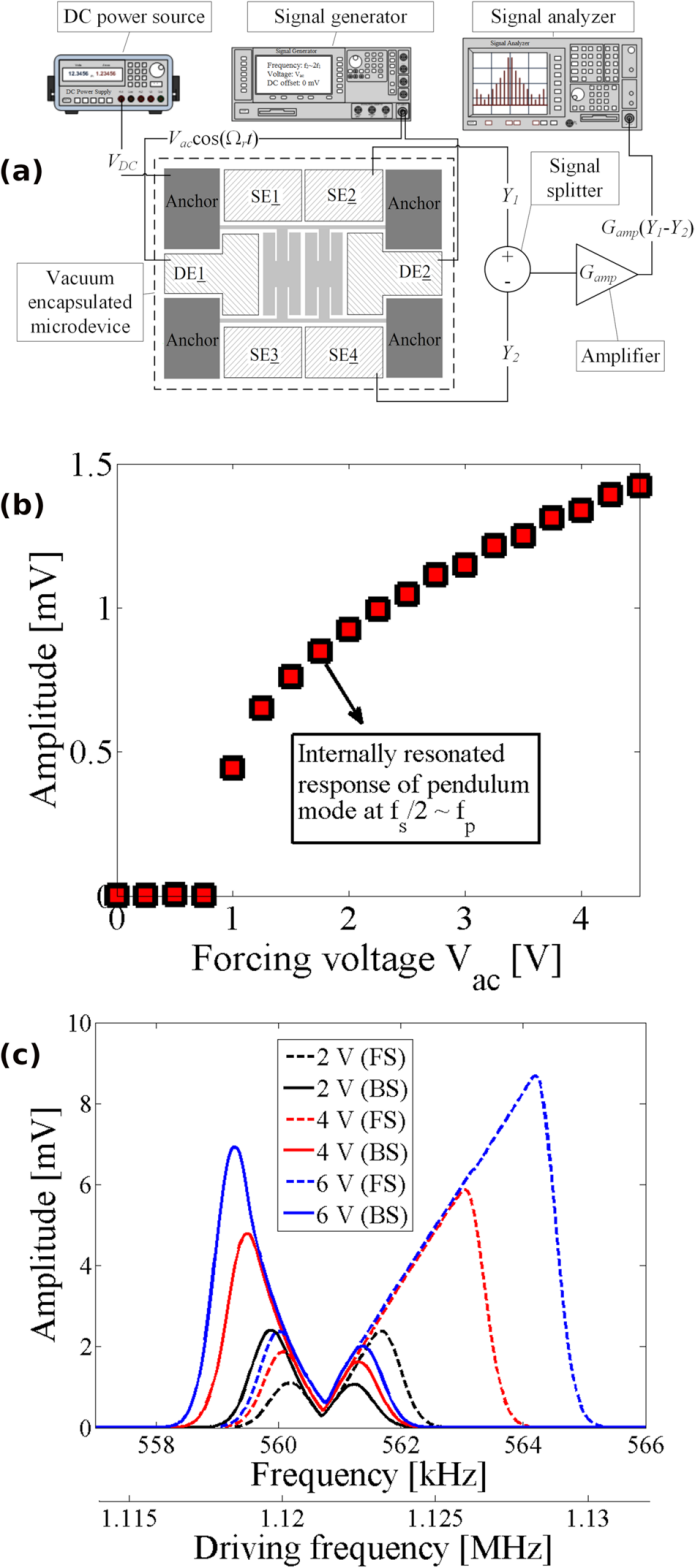
(**a**) The experimental setup to investigate saturation phenomenon and to perform frequency sweeps. (**b**) The measured pendulum-mode response versus the AC voltage amplitudes when ${{\rm{\Omega }}}_{r}=1120.\,4\,kHz\approx 2{f}_{p}$. (**c**) The nonlinear frequency transmission responses for three driving voltage amplitudes.

The nonlinear mode coupling caused by the 2:1 internal resonance is evaluated through the saturation figure and the nonlinear frequency response curves. The configuration in Fig. 3a was employed for these experiments where an AC voltage from a signal generator was superimposed on a DC voltage and used to produce the electrostatic input force. Output current due to the capacitance changes at the sense electrodes was differentially measured and monitored on a signal analyzer to allow for simultaneous monitoring of different portions of the spectrum. The intended 2:1 internal resonance and nonlinear mode coupling were examined through several experiments. Figure 3b demonstrates the half-order subharmonic response of the sense (i.e., pendulum) mode of the device as the drive voltage amplitude is increased and the frequency of excitation is fixed at Ω_*r*_ = 1120.45 *kHz* ≈ 2*f*_*p*_. As can be seen, the amplitude of the sense mode (i.e., pendulum mode) is negligible for drive voltage of less than *V*_*ac*_ ≤ ~1 *V*. However, once past this threshold voltage, the drive mode saturates and the additional energy is transferred to the sense mode for *V*_*ac*_ will result in more separation between the peaks. Figure 3c shows the nonlinear frequency response curves of the sense mode (i.e., pendulum mode) for three different actuation voltages. Despite the actuation of the system at the excitation frequency in the vicinity of the spring-mode resonant frequency, the pendulum mode responds at half the excitation frequency. As the electrostatic voltage is increased, the microresonator exhibit increasing nonlinear mode coupling, where the vibrational amplitude splits and two peaks of the vibrational deflection emerge in the vicinity of the pendulum-mode resonant frequency. Further increasing of the drive voltage, *V*_ac_, will result in additional separation between the peaks.

### Response to angular rate

**Figure 4:**
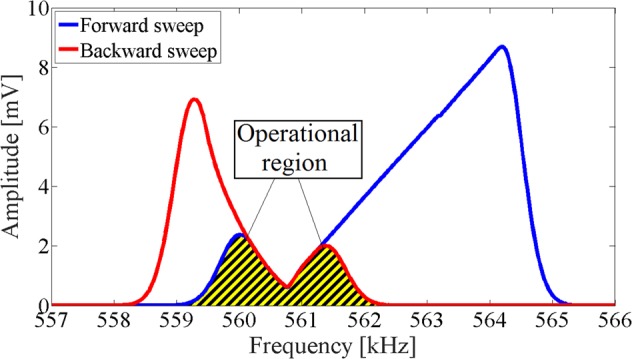
Operational frequency region for the microresonator when *V*_*ac*_ = 6 V and *V*_*DC*_ = 100 V.

For performance evaluation as a rate sensor, the overlap between the forward- and backward sweeps, as shown in Fig. 4, was selected as a robust region for applying the input force. To operate the sensor in this region, the frequency of the drive signal needs to be about twice some frequency in this band, eliminating the need for precise frequency control and providing a robust method for device excitation.

**Figure 5:**
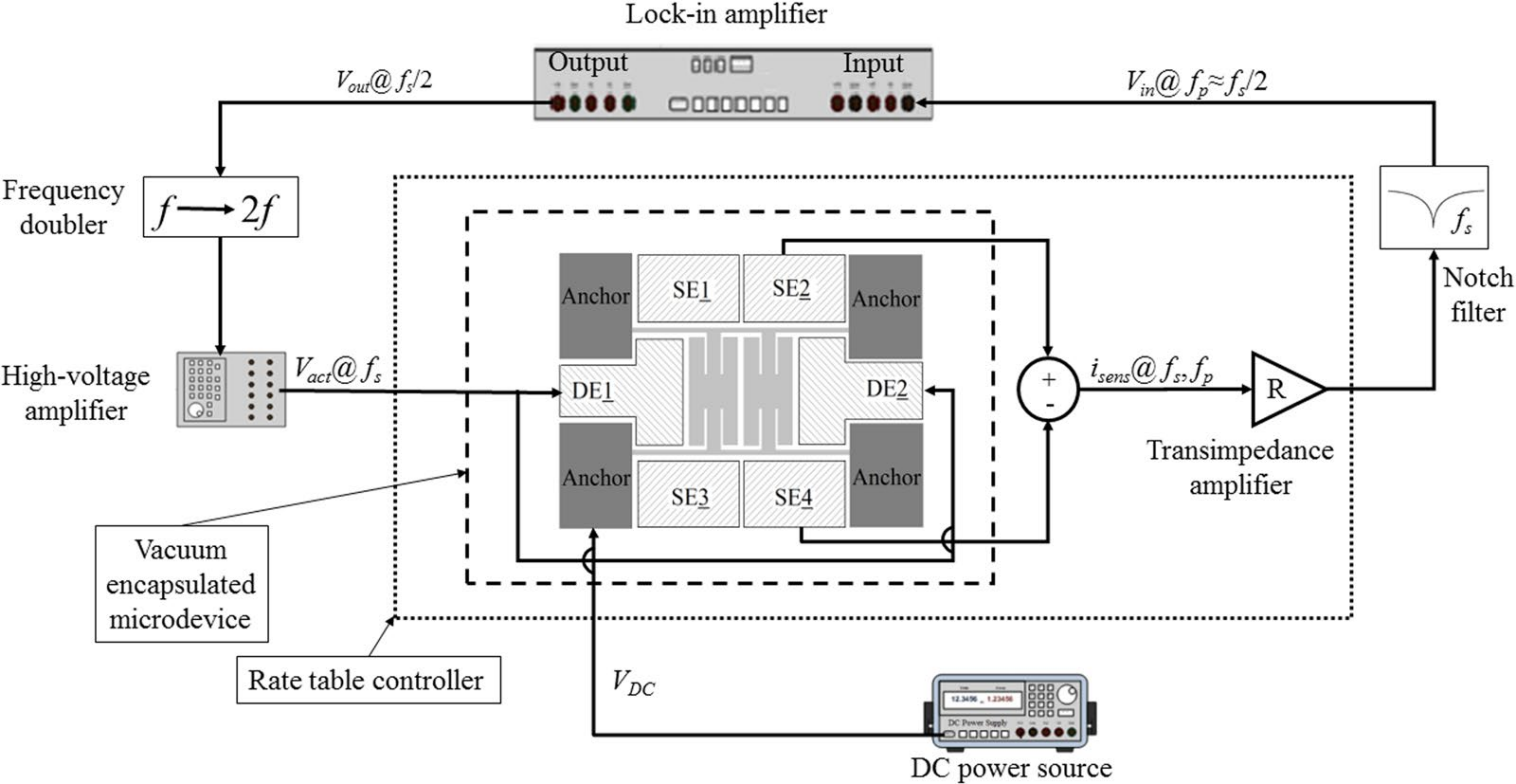
Block diagram of actuation and detection scheme used for characterization of the sensitivity to angular rate.

**Figure 6:**
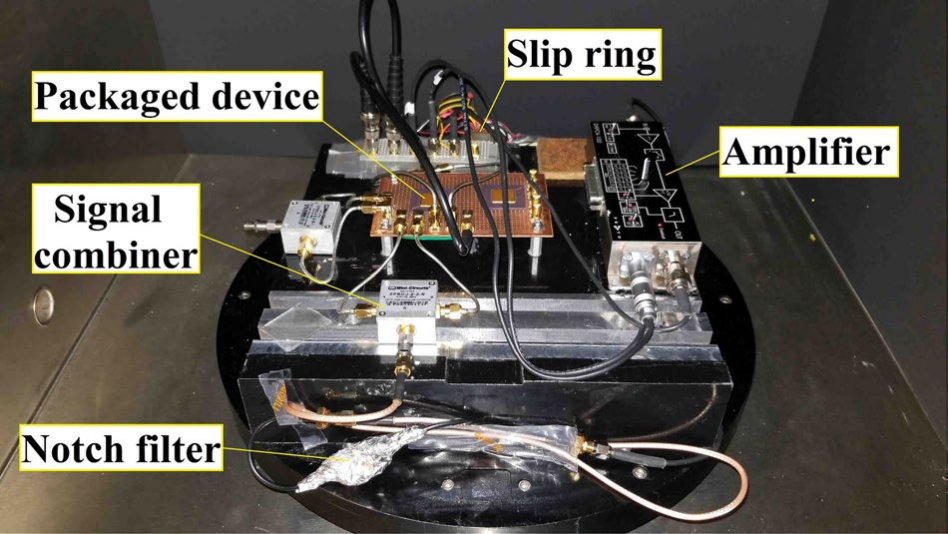
The photograph of the packaged device mounted on a circuit board with immediate electronics on the rate table.

**Figure 7:**
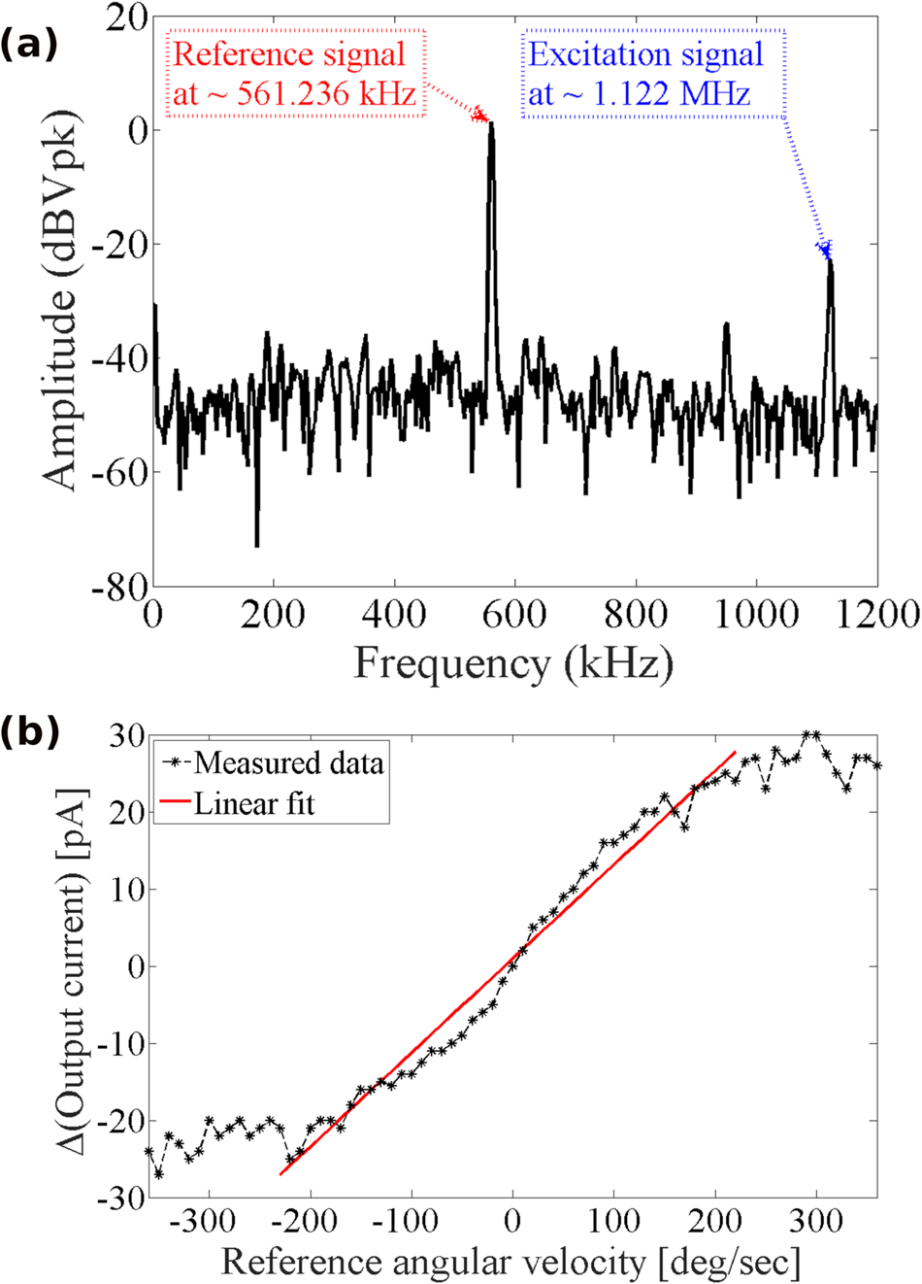
(**a**) Output spectrum of the device showing the reference signal (i.e., the detected sense amplitude prior to application of rata) and the parasitically coupled excitation signal (attenuated by the notch filter). (**b**) Measured sensitivity of the device in a range of ±360 deg + sec^−1^ with 10 deg + sec^−1^ steps.

To assess the scale factor (i.e., sensitivity) of device to input angular rate, the setup illustrated in Fig. 5 was used. The device chip was mounted inside a ceramic package and affixed to a printed circuit board (PCB). The PCB was then securely put on a rate table with a trans-resistance amplifier as shown in Fig. 6. The excitation signal was produced by a lock-in amplifier and passed through a frequency doubler before application to the sensor to excite the drive mode. Vibrations at the sense mode were detected by differential amplification of the currents from the sense electrodes and removing the interference from the excitation signal using a notch filter. The at-rest output spectrum of the microdevice is shown in Fig. 7a, confirming the activation of the nonlinear mode coupling due to the 2:1 internal resonance. Rate signals in the range of ±360 deg + sec^−1^ were applied to the device using the rate-table controller. Figure 7b shows the calibration curve obtained for different constant angular rates on the rate table and observing the corresponding output voltages of the microresonator. The *x*-axis in the figure represents the reference signal read from the rate-table controller while the *y*-axis represents the difference between the output current in response to the input rate and the at-rest signal from the resonator. Due to the quadratic nature of the nonlinearities in 2:1 internal resonance, the direction of the rate signal needs to be obtained from inertial signals. As seen in Fig. 7b, the microresonator demonstrates a sensitivity of 110 fA/(deg + sec^−1^) over a measurement range of approximately ±220 deg + sec^−1^ with a DC bias voltage of 80 V. At higher rates, the microresonator output exhibits reduced sensitivity to the input. This is likely due to the effect of centrifugal force on the device response which results in the detuning of mode frequency ratio, and hence, affecting the efficiency of energy exchange between the drive and sense modes.

## Discussion

There has been a significant level of interest in understanding the nonlinear dynamics of micro- and nano-structures. Except for the basic research efforts on the nonlinear phenomena, the understanding of nonlinearities was deemed necessary in order to devise methods to avoid them in practical applications. Herein, we presented a proof-of-concept design for a nonlinear rate microsensor that employs 2:1 internal resonance for its operation. In contrast to the typical designs for Coriolis vibratory gyroscopes with linearly coupled drive and sense modes, utilisation of internal resonance provides a robust means for excitation of the resonator over a range of input frequencies. For instance, in Fig. 4 it can be seen that the two vibration modes are coupled to each other effectively where the sense amplitude changes from about ~0.75 mV to ~2 mV over a sense frequency range of 559.5 kHz to 561.5 kHz. In case of a linear CVG with a resonant frequency of 561 kHz and similar quality factor of 4000, a 1 kHz deviation of drive signal would result in a drop in signal amplitude of ~14×. For this reason, the nonlinear operation scheme to a great extent alleviates the mode-tuning requirements for the drive and sense modes of the device. Additionally, the nonlinear operation of the device results in separation of the electrical drive and sense signals in the frequency domain, potentially simplifying the signal processing by removing direct interference between them. On the other hand, the nonlinear operation reduces the linear cross-coupling between the drive and sense directions that degrade the performance of linearly-coupled CVGs. This is specifically due to the fact that the linear cross-coupling terms will not force the system to resonate due to 2:1 frequency ratio between the spring and pendulum modes. Furthermore, their effect is negligible when compared to the quadratic couplings in the system.

As the first structural design to demonstrate the concept, the device offered a sensitivity of 110 fA/(deg + sec^−1^). The device sensitivity can improve, for example, by increasing the proof-masses used for the resonators. It is also possible to improve the device performance metrics such as sensitivity, dynamic range, and bandwidth through nonlinear closed-loop control. Further research will be needed to explore the limits of operation regarding noise performance or long-term stability of device response.

## Methods

### Fabrication process

The microresonator was fabricated through the *MEMS Integrated Design for Inertial Sensors* (MIDIS) process offered by Teledyne DALSA Inc.^13^. The MIDIS process is based on high aspect-ratio, bulk micromachining of a 30 μm thick single-crystal silicon wafer (*device layer*) that is sandwiched between two other silicon wafers (*top interconnect and bottom handle wafers*). The device can be either be vacuum encapsulated at 10 mTorr or held at a sub-atmospheric pressure of 150 Torr. The top silicon wafer includes Through Silicon Vias (TSV) with sealed anchors for compact flip-chip integration and interconnection with external microelectronic signal processing circuitry^28^. The encapsulation of vibrational inertial MEMS resonators at low pressures influences their quality factor and response time^28,29^. Figure 1a shows the top-view, Scanning Electron Microscope (SEM) image of the fabricated device before encapsulation with the top wafer. The dimensions of the fabricated device are *L*_*a*_ = 165 m, *w*_*a*_ = 8 m, *L*_*c*_ = 76 m, *L*_*tf*_ = 72 m, *w*_*tf*_ = 25 m, *w*_1_ = 29 m, *h*_1_ = 60 m, *w*_2_ = 35 m, *h*_2_ = 201 m, *l*_1_ = 194 m, *l*_2_ = 197 m, *g* = 1.75 μm, to achieve the approximate 2:1 ratio between the modes (Fig. 1b).

### Frequency domain characterisation

The measurement setup in Fig. 2a comprises (1) the vacuum encapsulated microresonator chip; (2) a DC power source (Keysight B2901A) to produce *V*_*DC*_; (3) a vector network analyzer (Rohde & Schwarz ZVB4) to produce ${V}_{ac}\,\cos ({{\rm{\Omega }}}_{r}t)$; (4) a signal combiner/splitter (Mini-Circuits ZFSCJ-2-2-S); and (5) a transimpedance amplifier (Zurich Instruments HF2TA) with a gain of *G*_*amp*_. In this figure, the signals *Y*_1_ and *Y*_2_ are the currents produced due to capacitance changes between the stationary sense electrodes SE2 and SE4 and the crossbars of the resonator. The setting to determine the natural frequencies and the Q-factors were *V*_*DC*_ = 100 *V*, *V*_*ac*_ = 1 *V*, and *G*_*amp*_ = 1 *k*Ω.

The nonlinear frequency measurement setup in Fig. 3a includes (1) the vacuum encapsulated microresonator chip; (2) a DC power source (Keysight B2901A) to produce *V*_*DC*_; (3) a function generator (Agilent Technologies 81150A) to produce *V*_*ac*_; (4) a signal combiner/splitter (Mini-Circuits ZFSCJ-2-2-S); (5) a transimpedance amplifier (Zurich Instruments HF2TA) with a gain of *G*_*amp*_; and (6) a signal analyzer (Agilent Technologies N9000A) to monitor the sensed signals in the frequency domain. For these tests, we set *V*_*DC*_ = 100 *V*, *V*_*ac*_ = 0–4.5 *V*, and *G*_*amp*_ = 10 *k*Ω. The drive frequency of the electrostatic voltage is then swept forward and backward around the spring mode frequency while monitoring the frequency response of the sense mode (pendulum mode).

### Sensitivity characterisation

The linearity, full-scale range, and response of the microresonator to the input rotation rate are extracted from the data obtained through the scale factor tests using a high precision rate table (Ideal Aerosmith 1621-200A-TL with AERO 812 controller). The schematic of the setup for the rate measurement is shown in Fig. 5. The microresonator is mounted on a PCB and securely placed on the rate table along with a low-noise trans-impedance amplifier (FEMTO DHPCA-100). A lock-in amplifier (Zurich Instruments HF2LI) was used to produce the excitation voltage and record the measured signals. While the lock-in amplifier could detect higher harmonics of the excitation signal, it is unable to capture subharmonics. On the other hand, the nonlinear transformation of the excitation signal from the frequency *f*_*s*_ to ${f}_{p}\approx {f}_{s}/2$ meant that the sense signal is roughly at half the frequency of the excitation signal, which would have been undetectable with the standard configuration. To circumvent this issue, a frequency doubler was used at the output of the lock-in amplifier to produce the excitation signal which was further amplified using a voltage amplifier (TEGAM 2350), and thus, treated as the first harmonic of the reference signal produced by the lock-in amplifier. The frequency doubler was designed using an analog multiplier chip (AD835) to multiply the reference signal by itself to generate its second harmonic at double the frequency. The signal at the output of the device included the sense signal at *f*_*p*_ due to the nonlinear excitation of the sense mode as well as a parasitic contribution from the drive signal at *f*_*s*_. The interference at *f*_*s*_ was then removed using a notch filter after initial amplification of the current signal using the transimpedance amplifier. The sense signal received by the lock-in amplifier was therefore at frequency *f*_*p*_, which was about *f*_*s*_/2.

An input rate in the form of a trapezoidal wave was then applied to the sensor such that the applied rate was increased from 0 deg + sec^−1^ to the target rate with the constant angular acceleration of 40 deg + sec^−2^, kept constant at the intended rate for 30 s, and then decreased back to 0 deg + sec^−1^ with the constant angular acceleration of −40 deg + sec^−2^. An accelerometer was used to determine the direction of rotation of the rate table. During the test, the output response of the microresonator is monitored and recorded. The response of the sensor is measured as the change in the amplitude of the sense signal due to the input rate.

For the sensitivity tests, we set *V*_DC_ = 0. 4 V at a frequency of 561. 236 kHz (before frequency doubler), a fixed gain of 20 V/V for the high-voltage amplifier, and a gain of *R* = 5 × 10^7^ Ω for the transimpedance amplifier. The notch filter was designed based on a passive twin-T structure for a notch frequency of 1120 kHz.

## Supplementary information

A Nonlinear Rate Microsensor utilising Internal Resonance: Supplementary Information
