# Supplementary material for: A Nonlinear Rate Microsensor utilising Internal Resonance
Source: Sci Rep. 2019 Jun 17;9:8648. doi: 10.1038/s41598-019-44669-3 (PMC6572818; doi:10.1038/s41598-019-44669-3)
Supplement: Supplementary file 1 — A Nonlinear Rate Microsensor utilising Internal Resonance: Supplementary Information [file 41598_2019_44669_MOESM1_ESM.docx]

A Nonlinear Rate Microsensor utilising Internal Resonance: Supplementary Information

**Atabak Sarrafan, Soheil Azimi, Farid Golnaraghi, and Behraad Bahreyni***

Simon Fraser University, Surrey, BC, Canada V3T 0A3

Corresponding author: [behraad@ieee.org](mailto:behraad@ieee.org)

# THEORY

## Governing equations

The lumped modeling of the device dynamics in nonlinear regime involves some assumptions;

1. According to the FEM modal analysis in CoventorWare^©^, rotation with respect to the anchor is allowed at either of the tuning fork beams ends. Thus, it makes sense to consider the tuning fork beams on each side of the device as a simply supported beam with an attached H-shaped proof mass in the middle.
2. Each anchor beam can be considered as a cantilever beam.
3. The left and right resonators are assumed to be decoupled.
4. The resonators can rotate with respect to the anchor, caused by large deformation of the suspension beams.
5. The tuning fork beams displace translationally with respect to the anchor beams attached to them.
6. The anchor beams are pinned to the fixed support to allow the rotation at the junction point.
7. The silicon anchors are assumed to be fixed and rigid during entire nonlinear operation of the microresonator. Accordingly, the planar motion of the microdevice can be modeled as a 4-DOF mechanism.

It is perceptible from the schematic that the motion of the microdevice with the masses $\text{M}_{\text{1}}$ and $\text{M}_{\text{2}}$ can be described by using four generalized coordinates $\text{q}_{\text{1}}\text{=}\text{θ}_{\text{1}}\text{, }\text{q}_{\text{2}}\text{=}\text{r}_{\text{1}}\text{, }\text{q}_{\text{3}}\text{=}\text{θ}_{\text{2}}$ $\text{and }\text{q}_{\text{4}}\text{=}\text{r}_{\text{2}}$. The variables $\text{r}_{\text{1}}$ and $\text{r}_{\text{2}}$ describe the extensional motions of the masses $\text{M}_{\text{1}}$ and $\text{M}_{\text{2}}$, respectively. The generalized coordinates $\text{θ}_{\text{1}}$ and $\text{θ}_{\text{2}}$ represent the angular motions of masses $\text{M}_{\text{1}}$ and $\text{M}_{\text{2}}$ along with $\text{M}_{\text{b}}$, respectively. The (*X*, *Y*, *Z*) reference frame is stationary and centered at the point *O*. The mass $\text{M}_{\text{i}}$ represents the mass of the beam of length $\text{L}_{\text{tf}}$ with the central H-like proof mass. The mass $\text{M}_{\text{b}}$ represents the effective mass of two cantilever beams of length $\text{L}_{\text{e}}$ where $\text{L}_{\text{e}}\text{=}\text{L}_{\text{a}}\text{-0.5}\text{w}_{\text{tf}}$. The linear and rotational springs and dampers are considered to be present in both spring and pendulum motions. The electrostatic forces $\text{F}_{\text{1}}$ and $\text{F}_{\text{3}}$ are applied to the masses $\text{M}_{\text{1}}$ and $\text{M}_{\text{2}}$, respectively. The capacitive forces $\text{F}_{\text{2}}$ and $\text{F}_{\text{4}}$ stand for the difference between the electrostatic forces imposed on the device by the sense electrodes SE 1 and SE 3, and SE 2 and SE 4, respectively. The constant input angular rate is indicated by $\text{Ω}_{\text{Z}}$.

Consider the planar motion of the lumped-spring-damper model representing the microresonator (Figure S1).

| 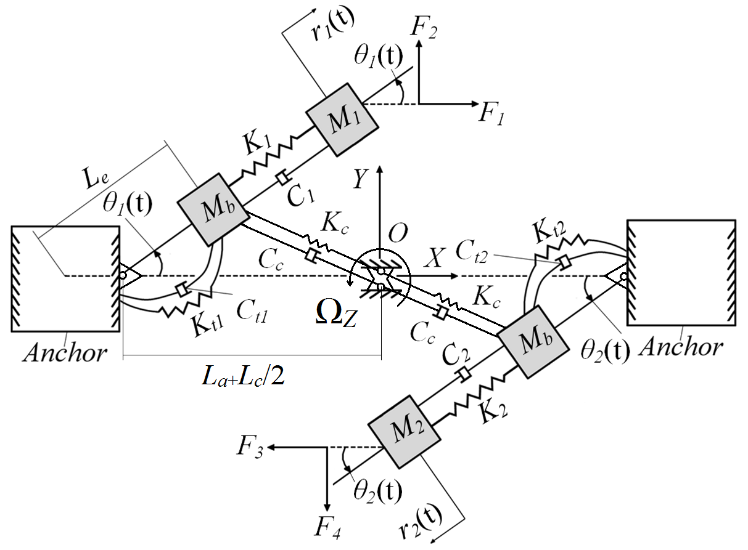 |
| --- |
| Figure S1. Lumped model of the coupled resonator |

Using Lagrange’s equation, we get the equations of motion as

| $\text{L}_{\text{e}}^{\text{2}}\left( \text{M}_{\text{b}}\text{+M}_{\text{1}} \right){\ddot{\text{θ}}}_{\text{1}}\left( \text{t} \right)\text{+C}_{\text{t1}}{\dot{\text{θ}}}_{\text{1}}\left( \text{t} \right)\text{+K}_{\text{t1}}\text{θ}_{\text{1}}\left( \text{t} \right)\text{=}\frac{\text{F}_{\text{2}}}{\cos\left( \text{θ}_{\text{1}}\left( \text{t} \right) \right)}\left( \text{L}_{\text{e}}\text{+r}_{\text{1}}\left( \text{t} \right) \right)$  $\text{-M}_{\text{1}}\left( \left( \text{r}_{\text{1}}^{\text{2}}\left( \text{t} \right){\text{+2L}_{\text{e}}\text{r}}_{\text{1}}\left( \text{t} \right) \right){\ddot{\text{θ}}}_{\text{1}}\left( \text{t} \right)\text{+2}\left( \text{L}_{\text{e}}\text{+r}_{\text{1}}\left( \text{t} \right) \right){\dot{\text{r}}}_{\text{1}}\left( \text{t} \right){\dot{\text{θ}}}_{\text{1}}\left( \text{t} \right) \right)$  $\text{-L}_{\text{e}}^{\text{2}}\left( \text{K}_{\text{c}}\frac{\sin\left( \text{θ}_{\text{1}}\left( \text{t} \right) \right)}{\text{cos}^{\text{3}}\left( \text{θ}_{\text{1}}\left( \text{t} \right) \right)}\text{+C}_{\text{c}}\frac{{\dot{\text{θ}}}_{\text{1}}\left( \text{t} \right)}{\text{cos}^{\text{4}}\left( \text{θ}_{\text{1}}\left( \text{t} \right) \right)} \right)$ | (1) |
| --- | --- |
| $\text{M}_{\text{1}}{\ddot{\text{r}}}_{\text{1}}\left( \text{t} \right){\dot{\text{+C}_{\text{1}}\text{r}}}_{\text{1}}\left( \text{t} \right){\text{+K}_{\text{1}}\text{r}}_{\text{1}}\left( \text{t} \right){\dot{\text{=M}_{\text{1}}\left( \text{L}_{\text{e}}\text{+r}_{\text{1}}\left( \text{t} \right) \right)\text{θ}}}_{\text{1}}^{\text{2}}\left( \text{t} \right)\text{+}\frac{\text{F}_{\text{1}}}{\cos\left( \text{θ}_{\text{1}}\left( \text{t} \right) \right)}$ | (2) |

where

$\text{F}_{\text{1}}\text{=}\frac{\text{1}}{\text{2}}\left( \text{V}_{\text{DC}}\text{+V}_{\text{ac}}\cos\left( \text{Ω}_{\text{r}}\text{t} \right) \right)^{\text{2}}\text{l}_{\text{1}}\frac{\text{ε}_{\text{0}}\text{t}_{\text{Si}}}{\left( \text{g-r}_{\text{1}}\left( \text{t} \right) \right)^{\text{2}}}$,

${\text{F}_{\text{2}}\text{=}\frac{\text{1}}{\text{2}}\text{V}}_{\text{DC}}^{\text{2}}\text{ε}_{\text{0}}\text{t}_{\text{Si}}\text{L}_{\text{e}}\text{l}_{\text{2}}\left( \text{1+}\left( \tan\left( \text{θ}_{\text{1}}\left( \text{t} \right) \right) \right)^{\text{2}} \right)\text{ }\left( \frac{\text{1}}{\left( \text{g-L}_{\text{e}}\tan\left( \text{θ}_{\text{1}}\left( \text{t} \right) \right) \right)^{\text{2}}}\text{+}\frac{\text{1}}{\left( \text{g+L}_{\text{e}}\tan\left( \text{θ}_{\text{1}}\left( \text{t} \right) \right) \right)^{\text{2}}} \right)$,

$\text{M}_{\text{1}}\text{=M}_{\text{Proof mass}}\text{+M}_{\text{tf beam}}{\text{=}\text{t}}_{\text{Si}}\text{ρ}_{\text{Si}}\left( {\text{2}\text{h}}_{\text{2}}\text{w}_{\text{2}}{\text{+}\text{h}}_{\text{1}}\text{w}_{\text{1}}\text{+L}_{\text{tf}}\text{w}_{\text{tf}} \right)$,

$\text{M}_{\text{b}}{\text{=0.4714 }\text{t}}_{\text{Si}} \text{ρ}_{\text{Si}} \text{L}_{\text{e}} \text{w}_{\text{a}}$,

$\text{f}_{\text{1}}\text{=}\left( \left( {\text{K}_{\text{t1}}\text{+K}_{\text{c}}\text{L}}_{\text{e}}^{\text{2}} \right)/{\text{L}_{\text{e}}^{\text{2}}\left( \text{M}_{\text{1}}\text{+M}_{\text{b}} \right)} \right)^{\text{1}/\text{2}}=\left( {\text{K}_{\text{t1eff}}}/{\text{L}_{\text{e}}^{\text{2}}\left( \text{M}_{\text{1}}\text{+M}_{\text{b}} \right)} \right)^{\text{1}/\text{2}}$,

${\text{ f}_{\text{2}}\text{=}\left( {\text{K}_{\text{1}}}/{\text{M}_{\text{1}}} \right)}^{\text{1}/\text{2}}$.

where $\varepsilon_{0}$ is the permittivity of the space ($8.85\times{10}^{-12}$ (F/m)); $t_{Si}$ is the thickness of Silicon device layer; $V_{DC}$ is the DC bias voltage applied to the sense and drive electrodes; $\rho_{Si}$ is the mass density of Silicon layer (2300 Kg/m^3^); $V_{ac}$ is the amplitude of the AC driving voltage applied to the drive electrodes, and $\Omega_{r}$ is the excitation frequency. The $Q_{i}$ stands for the quality factor corresponding to the i^th^-mode, $i=1$ and $2$. The translational $C_{1}$ and $C_{2}$ and rotational $C_{t1}$ and $C_{t2}$ damping coefficients are estimated using Rayleigh’s damping equation. The linearized natural frequencies of pendulum and spring modes are defined by $f_{1}$, $f_{2}$, $f_{3}$ and $f_{4}$, respectively.

## Perturbation analysis

The following equations represent the scaled and nondimensionalized equations of motion

| $\left. \begin{matrix} {\ddot{\text{ψ}}}_{\text{1}}\text{+}\text{ω}_{\text{1}}^{\text{2}}\text{ψ}_{\text{1}}\text{+ε}\left( \begin{aligned} {\text{2}\text{m}_{\text{1}}\dot{\text{ρ}}}_{\text{1}}{\dot{\text{ψ}}}_{\text{1}}\text{+2}\text{m}_{\text{1}}\text{ρ}_{\text{1}}{\ddot{\text{ψ}}}_{\text{1}}\text{+}\text{μ}_{\text{t1}}{\dot{\text{ψ}}}_{\text{1}} \\ \text{+2}\ddot{\text{φ}}\text{m}_{\text{1}}\text{ρ}_{\text{1}}\text{-}\ddot{\text{φ}}\text{m}_{\text{1}}\text{ρ}_{\text{1}}\frac{\left( \text{L}_{\text{a}}\text{+}\frac{\text{L}_{\text{c}}}{\text{2}} \right)}{\text{L}_{\text{e}}}\text{+2}\text{m}_{\text{1}}{\dot{\text{ρ}}}_{\text{1}}\dot{\text{φ}} \end{aligned} \right) \\ \text{+}\left( \text{1-}\frac{\left( \text{L}_{\text{a}}\text{+}\frac{\text{L}_{\text{c}}}{\text{2}} \right)}{\text{L}_{\text{e}}} \right)\ddot{\text{φ}}\text{+}\text{ε}^{\text{2}}\text{m}_{\text{1}}\left( \begin{aligned} \text{ρ}_{\text{1}}^{\text{2}}{\ddot{\text{φ}}\text{+ρ}}_{\text{1}}\left( \text{ρ}_{\text{1}}{\ddot{\text{ψ}}}_{\text{1}}\text{+2}{\dot{\text{ρ}}}_{\text{1}}{\dot{\text{ψ}}}_{\text{1}} \right) \\ \text{+2}{\dot{\text{ρ}}}_{\text{1}}\text{ρ}_{\text{1}}\dot{\text{φ}}\text{-}\frac{\left( \text{L}_{\text{a}}\text{+}\frac{\text{L}_{\text{c}}}{\text{2}} \right)}{\text{L}_{\text{e}}}\text{ψ}_{\text{1}}{\dot{\text{φ}}}^{\text{2}} \end{aligned} \right) \\ \begin{matrix} \text{=}\text{p}_{\text{0}}\text{t}_{\text{Si}}\frac{\text{l}_{\text{2}}}{\left( \text{M}_{\text{1}}\text{+}\text{M}_{\text{b}} \right)\text{Ω}^{\text{2}}\text{g}^{\text{2}}}\text{V}_{\text{dc}}^{\text{2}}\left( \text{ρ}_{\text{1}}\text{ε}^{\text{2}}\text{+ε} \right) \\ \begin{matrix} {\ddot{\text{ρ}}}_{\text{1}}\text{+}\text{ω}_{\text{2}}^{\text{2}}\text{ρ}_{\text{1}}\text{+ε}\left( \text{μ}_{\text{1}}{\dot{\text{ρ}}}_{\text{1}}\text{-}{\dot{\text{ψ}}}_{\text{1}}^{\text{2}}\text{-}\frac{\left( \text{L}_{\text{a}}\text{+}\frac{\text{L}_{\text{c}}}{\text{2}} \right)}{\text{L}_{\text{e}}}\text{ψ}_{\text{1}}\ddot{\text{φ}}\text{+}\left( \frac{\left( \text{L}_{\text{a}}\text{+}\frac{\text{L}_{\text{c}}}{\text{2}} \right)}{\text{L}_{\text{e}}}\text{-1} \right){\dot{\text{φ}}}^{\text{2}}\text{-2}{\dot{\text{ψ}}}_{\text{1}}\dot{\text{φ}} \right) \end{matrix} \\ \begin{matrix} \text{-}\text{ε}^{\text{2}}\left( \text{2ρ}_{\text{1}}\dot{\text{φ}{\dot{\text{ψ}}}_{\text{1}}}\text{+}\text{ρ}_{\text{1}}{\dot{\text{ψ}}}_{\text{1}}^{\text{2}}\text{+}\text{ρ}_{\text{1}}{\dot{\text{φ}}}^{\text{2}} \right) \\ \text{=}\text{p}_{\text{0}}\text{t}_{\text{Si}}\text{l}_{\text{1}}\left( \text{V}_{\text{dc}}\text{+}\text{V}_{\text{AC}}\text{cos}\left( \text{Ω}_{\text{1}}\text{τ} \right) \right)^{\text{2}}\left( \frac{{\text{ε}^{\text{2}}\text{ρ}}_{\text{1}}}{\text{M}_{\text{1}}\text{g}^{\text{3}}\text{Ω}^{\text{2}}}\text{+}\frac{\text{ε}}{\text{2}\text{M}_{\text{1}}{\text{L}_{\text{e}}\text{g}}^{\text{2}}\text{Ω}^{\text{2}}} \right) \end{matrix} \end{matrix} \end{matrix} \right\}$ |
| --- |

where

| $\text{r}_{\text{1}}\left( \text{t} \right)\text{=}\text{Ρ}_{\text{1}}\left( \text{τ} \right)\text{L}_{\text{e}}\text{, }\text{θ}_{\text{1}}\left( \text{t} \right)\text{=}\text{Θ}_{\text{1}}\left( \text{τ} \right), \text{t =}\frac{\text{τ}}{\text{Ω}}\text{, }\frac{\text{d}}{\text{dt}}\text{=Ω}\frac{\text{d}}{\text{dτ}}\text{, }\frac{\text{d}^{\text{2}}}{\text{dt}^{\text{2}}}\text{=}\text{Ω}^{\text{2}}\frac{\text{d}^{\text{2}}}{\text{dτ}^{\text{2}}}$  $\text{Θ}_{\text{1}}\left( \text{τ} \right)\text{=ε}\text{ψ}_{\text{1}}\left( \text{τ} \right)\text{, }\text{Ρ}_{\text{1}}\left( \text{τ} \right)\text{=ε}\text{ρ}_{\text{1}}\left( \text{τ} \right)\text{, }\text{Ω}_{\text{Z}}\text{=ε}\dot{\text{φ}}\left( \text{τ} \right)\text{, }\text{V}_{\text{ac}}\text{=ε}\text{V}_{\text{AC}}\text{, }\text{V}_{\text{DC}}\text{=ε}\text{V}_{\text{dc}}\text{, }\text{γ}_{\text{t1}}\text{= ε}\text{μ}_{\text{t1}}\text{, }\text{γ}_{\text{1}}\text{= ε}\text{μ}_{\text{1}}$  $\text{ω}_{\text{1}}\text{=}\frac{\sqrt{\frac{\text{K}_{\text{t1}}\text{+}\text{K}_{\text{c}}\text{L}_{\text{e}}^{\text{2}}}{\left( \text{M}_{\text{1}}\text{+}\text{M}_{\text{b}} \right)\text{L}_{\text{e}}^{\text{2}}}}}{\text{Ω}}\text{, }\text{ω}_{\text{2}}\text{=}\frac{\sqrt{\frac{\text{K}_{\text{1}}}{\text{M}_{\text{1}}}}}{\text{Ω}}\text{, }\text{m}_{\text{1}}\text{=}\frac{\text{M}_{\text{1}}}{\text{M}_{\text{1}}\text{+}\text{M}_{\text{b}}}\text{, }\text{Ω}_{\text{1}}\text{=}\frac{\text{Ω}_{\text{r}}}{\text{Ω}}, \text{γ}_{\text{t1}}\text{=}\frac{\text{C}_{\text{t1}}\text{+}\text{C}_{\text{c}}\text{L}_{\text{e}}^{\text{2}}}{\left( \text{M}_{\text{1}}\text{+}\text{M}_{\text{b}} \right)\text{L}_{\text{e}}^{\text{2}}\text{Ω}}\text{=}\frac{\text{C}_{\text{t1eff}}}{\left( \text{M}_{\text{1}}\text{+}\text{M}_{\text{b}} \right)\text{L}_{\text{e}}^{\text{2}}\text{Ω}}\text{,}\text{γ}_{\text{1}}\text{=}\frac{\text{C}_{\text{1}}}{\text{M}_{\text{1}}\text{Ω}}$ |
| --- |

In the above equation, $\text{Ω}$ is the nondimensionalizing frequency, and $\text{ε}$ is a small dimensionless parameter, 0$<\text{ε}\text{≪}\text{1}$, expressing the order of nonlinearity and coupling. The method of two-variable expansion is used to obtain approximate solution for the equations (1) and (2) in case of the external resonance ($\Omega_{1} \cong\omega_{2}$) and the internal resonance ($\omega_{2}\cong2\omega_{1}$). To describe how close the frequencies are to the resonance conditions we introduce detuning parameters:

|  | $\Omega_{1}=\omega_{2}+\varepsilon\sigma_{1}, {{\text{ ω}_{\text{2}}\text{=2ω}}_{\text{1}}\text{+εσ}}_{\text{2}}$ | (3) |
| --- | --- | --- |

where $\sigma_{1}$ and $\sigma_{2}$ are called the external and internal detuning parameters, respectively. The solvability conditions (the conditions for the elimination of secular terms) can be written as

|  | $\left. \begin{matrix} \text{α}_{\text{1η}}\text{=}\frac{\text{1}}{\text{2}}\text{m}_{\text{1}}\text{a}_{\text{2}}\left( \text{ω}_{\text{1}}\text{-ω}_{\text{2}} \right)\cos\left( \text{β}_{\text{1}} \right) \\ \text{a}_{\text{1η}}\text{=-}\frac{\text{μ}_{\text{t1}}}{\text{2}}\text{a}_{\text{1}}\text{+}\frac{\text{1}}{\text{2}}\text{m}_{\text{1}}\left( \text{ω}_{\text{1}}\text{-ω}_{\text{2}} \right)\text{a}_{\text{1}}\text{a}_{\text{2}}\sin\left( \text{β}_{\text{1}} \right) \\ \begin{matrix} {\alpha_{\text{2η}}\text{=-}\frac{\text{ω}_{\text{1}}^{\text{2}}}{\text{4a}_{\text{2}}\text{ω}_{\text{2}}}\text{a}}_{\text{1}}^{\text{2}}\cos\left( \text{β}_{\text{1}} \right)\text{+}\frac{\text{ε}_{\text{0}}\text{t}_{\text{Si}}\text{l}_{\text{1}}}{\text{2M}_{\text{1}}\text{L}_{\text{e}}\left( \text{g}\text{Ω} \right)^{\text{2}}\text{ω}_{\text{2}}\text{a}_{\text{2}}}\text{V}_{\text{DC}}\text{V}_{\text{AC}}\cos\left( \text{β}_{\text{2}} \right) \\ \text{a}_{\text{2η}}\text{=-}\frac{\text{μ}_{\text{1}}}{\text{2}}\text{a}_{\text{2}}\text{+}\frac{\text{ω}_{\text{1}}^{\text{2}}}{\text{4ω}_{\text{2}}}\text{a}_{\text{1}}^{\text{2}}\text{sin} \left( \text{β}_{\text{1}} \right)\text{+}\frac{\text{ε}_{\text{0}}\text{t}_{\text{Si}}\text{l}_{\text{1}}}{\text{2M}_{\text{1}}\text{L}_{\text{e}}\left( \text{gΩ} \right)^{\text{2}}\text{ω}_{\text{2}}}\text{V}_{\text{DC}}\text{V}_{\text{AC}}\sin\left( \text{β}_{\text{2}} \right) \end{matrix} \end{matrix} \right\}$ | (4) |
| --- | --- | --- |

where

|  | $\text{β}_{\text{1}}\text{=2α}_{\text{1}}\text{-α}_{\text{2}}\text{+σ}_{\text{2}}\text{η , }\text{β}_{\text{2}}\text{= }\text{α}_{\text{2}}\text{+σ}_{\text{1}}\text{η}$ | (5) |
| --- | --- | --- |

To obtain the steady-state solutions, solving $a_{1\eta}=a_{2\eta}=\beta_{1\eta}=\beta_{2\eta}=0$, there are two possible cases for equation (5). The first case implies the solutions for linear systems, as follows

|  | $\text{ψ}_{\text{1}}\text{= 0}, \text{ρ}_{\text{1}}\text{=}\frac{\text{ε}_{\text{0}}\text{t}_{\text{Si}}\text{l}_{\text{1}}\text{V}_{\text{DC}}\text{V}_{\text{AC}}}{\text{M}_{\text{1}}\text{ω}_{\text{2}}\text{L}_{\text{e}}\left( \text{gΩ} \right)^{\text{2}}\sqrt{{\text{4σ}_{\text{1}}^{\text{2}}\text{+μ}}_{\text{1}}^{\text{2}}}}\cos\left( \text{Ω}_{\text{1}}\text{τ-}\text{tan}^{\text{-1}} \left( \frac{\text{μ}_{\text{1}}}{\sqrt{{\text{4σ}_{\text{1}}^{\text{2}}\text{+μ}}_{\text{1}}^{\text{2}}}}-\frac{\text{2σ}_{\text{1}}}{\sqrt{{\text{4σ}_{\text{1}}^{\text{2}}\text{+μ}}_{\text{1}}^{\text{2}}}} \right) \right)$ | (6) |
| --- | --- | --- |

The second case is:

|  | $\left. \begin{matrix} \text{a}_{\text{1}}\text{=-}\frac{\text{1}}{\text{ω}_{\text{1}}\text{gΩ}}\left[ \frac{\text{2}\left( \text{Γ}_{\text{1}}\text{±}\sqrt{\left( \text{Γ}_{\text{2}}\text{+Γ}_{\text{3}} \right)\left( \text{Γ}_{\text{2}}\text{-Γ}_{\text{3}} \right)} \right)}{\text{M}_{\text{1}}\text{m}_{\text{1}}\text{L}_{\text{e}}\left( \text{ω}_{\text{1}}\text{-ω}_{\text{2}} \right)} \right]^{\frac{\text{1}}{\text{2}}} \\ \text{a}_{\text{2}}\text{=}\frac{\sqrt{\left( \text{μ}_{\text{t1}} \right)^{\text{2}}\text{+}\left( \text{σ}_{\text{1}}\text{+σ}_{\text{2}} \right)^{\text{2}}}}{\text{m}_{\text{1}}\left( \text{ω}_{\text{2}}\text{-ω}_{\text{1}} \right)} \\ \begin{matrix} \text{β}_{\text{1}}\text{=}\text{tan}^{\text{-1}} \left( \text{-}\frac{\text{μ}_{\text{t1}}}{\sqrt{\left( \text{μ}_{\text{t1}} \right)^{\text{2}}\text{+}\left( \text{σ}_{\text{1}}\text{+σ}_{\text{2}} \right)^{\text{2}}}},\frac{\text{σ}_{\text{1}}\text{+σ}_{\text{2}}}{\sqrt{\left( \text{μ}_{\text{t1}} \right)^{\text{2}}\text{+}\left( \text{σ}_{\text{1}}\text{+σ}_{\text{2}} \right)^{\text{2}}}} \right) \\ \text{β}_{\text{2}}\text{=}\text{-}\text{tan}^{\text{-1}} \left( \frac{\left( \text{σ}_{\text{1}}\text{+σ}_{\text{2}} \right)\text{Γ}_{\text{3}}\text{-μ}_{\text{t1}}\sqrt{\left( \text{Γ}_{\text{2}}\text{+Γ}_{\text{3}} \right)\left( \text{Γ}_{\text{2}}\text{-Γ}_{\text{3}} \right)}}{\text{μ}_{\text{t1}}\text{Γ}_{\text{3}}\text{+}\left( \text{σ}_{\text{1}}\text{+σ}_{\text{2}} \right)\sqrt{\left( \text{Γ}_{\text{2}}\text{+Γ}_{\text{3}} \right)\left( \text{Γ}_{\text{2}}\text{-Γ}_{\text{3}} \right)}} \right) \end{matrix} \end{matrix} \right\}$ | (7) |
| --- | --- | --- |

where

$\text{Γ}_{\text{1}}\text{=}{\text{M}_{\text{1}}\text{ω}_{\text{2}}\text{L}_{\text{e}}\left( \text{gΩ} \right)}^{\text{2}}\left( \text{μ}_{\text{1}}\text{μ}_{\text{t1}}\text{-2σ}_{\text{1}}\left( \text{σ}_{\text{1}}{\text{+}\text{σ}}_{\text{2}} \right) \right)$,

$\Gamma_{2} =\varepsilon_{0} t_{\mathrm{Si}}V_{\mathrm{DC}} V_{\mathrm{AC}} \left( \omega_{1} -\omega_{2} \right) m_{1} l_{1}$,

$\text{Γ}_{\text{3}}\text{=}{\text{M}_{\text{1}}\text{ω}_{\text{2}}\text{L}_{\text{e}}\left( \text{gΩ} \right)}^{\text{2}}\left( \left( \text{μ}_{\text{1}}\text{+2μ}_{\text{t1}} \right)\text{σ}_{\text{1}}\text{+μ}_{\text{1}}\text{σ}_{\text{2}} \right)$.

The steady-state response for this case are

|  | $\left. \begin{matrix} \text{ψ}_{\text{1}}\text{=-}\frac{\text{1}}{\text{ω}_{\text{1}}\text{gΩ}}\left[ \frac{\text{2}\left( \text{Γ}_{\text{1}}\text{±}\sqrt{\left( \text{Γ}_{\text{2}}\text{+Γ}_{\text{3}} \right)\left( \text{Γ}_{\text{2}}\text{-Γ}_{\text{3}} \right)} \right)}{\text{M}_{\text{1}}\text{m}_{\text{1}}\text{L}_{\text{e}}\left( \text{ω}_{\text{1}}\text{-ω}_{\text{2}} \right)} \right]^{\frac{\text{1}}{\text{2}}}\text{cos} \left( \frac{\text{1}}{\text{2}}\left( \text{Ω}_{\text{1}}\text{τ-β}_{\text{1}}\text{-β}_{\text{2}} \right) \right)\text{+ O}\left( \text{ε} \right) \\ \text{ρ}_{\text{1}}\text{=}\frac{\sqrt{\left( \text{μ}_{\text{t1}} \right)^{\text{2}}\text{+}\left( \text{σ}_{\text{1}}\text{+σ}_{\text{2}} \right)^{\text{2}}}}{\text{m}_{\text{1}}\left( \text{ω}_{\text{2}}\text{-ω}_{\text{1}} \right)}\text{cos} \left( \text{Ω}_{\text{1}}\text{τ-β}_{\text{2}} \right)\text{+O}\left( \text{ε} \right) \end{matrix} \right\}$ | (8) |
| --- | --- | --- |

The solutions for the second case correspond to the nonlinear behavior of the system when the nonlinear internal resonance occurs. The system parameters utilized for simulation can be found in Table S1.

| Experiment  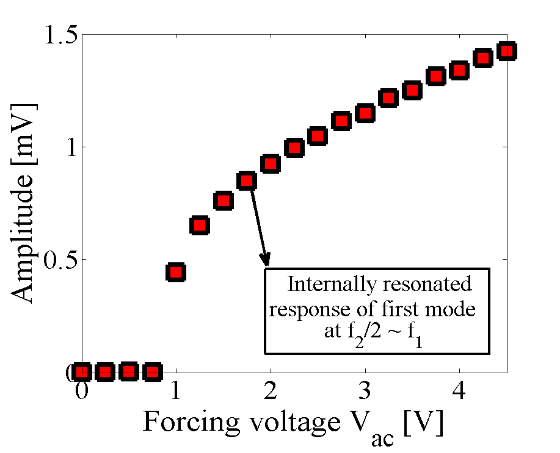  (a) | Simulation  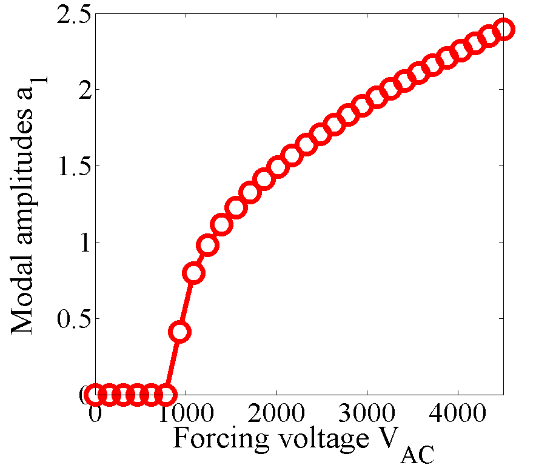  (b) |
| --- | --- |

Figure S2. The pendulum-mode response in the H-shaped microresonator versus the AC voltage amplitudes. (a) Experimentally measured when $Ω_{r}=2f_{1}\approx1$.120448 MHz and (b) Simulated response when $\sigma_{1}=-\sigma_{2}$ or $Ω_{1}=2\omega_{1}$.

Table S1. System parameter values for the H-shaped microresonator.

| **Symbol** | **Value** |  | **Symbol** | **Value** |
| --- | --- | --- | --- | --- |
| $\text{ω}_{\text{1}}$ | 1 |  | $\text{M}_{\text{1}}$ | $\text{1.32}\text{×}\text{10}^{\text{-9}}$ Kg |
| $\text{ω}_{\text{2}}$ | 2.001 |  | $\text{C}_{\text{1}}$ | $\text{1.63}\text{×}\text{10}^{\text{-6}}$ N/m.sec |
| $\text{Ω}$ | $\text{ω}_{\text{n}_{\text{1}}}$ |  | $\text{C}_{\text{t1eff}}$ | $\text{1.62}\text{×}\text{10}^{\text{-14}}$ N.m/sec |
| $\text{m}_{\text{1}}$ | 0.97 |  | $\text{K}_{\text{1}}$ | $\text{6.55}\text{×}\text{10}^{\text{4}}$ N/m |
| $\text{μ}_{\text{1}}$ | 0.35 |  | $\text{K}_{\text{t1eff}}$ | $\text{3.93}\text{×}\text{10}^{\text{-4}}$ N.m |
| $\text{μ}_{\text{t1}}$ | 0.14 |  | $\text{M}_{\text{b}}$ | $\text{4.31}\text{×}\text{10}^{\text{-11}}$ Kg |
| $\text{ε}$ | 0.001 |  | $\text{L}_{\text{e}}$ | 152 μm |
| $\text{σ}_{\text{2}}$ | 1 |  | g | 1.75 μm |

# EXPERIMENTAL AND ANALYTICAL SIMULATION RESULTS

Multiple experiments were performed to reveal the intended 2:1 internal resonance and nonlinear mode coupling in the microresonator. Figure S2 exhibits the experimental and simulated half-order subharmonic response of the pendulum mode of the device as the drive voltage amplitude is increased and the frequency of excitation is fixed at $f_{2}\approx2f_{1}$ (experiment) or $\omega_{2}\approx2 \omega_{1}$ (simulation). For tests, we set $V_{DC}=100V$, $V_{AC}=0-4.5 V$ and $G_{amp}=10 kΩ$. Figure S2 shows the amplitude of the spike generated at half the excitation frequency (i.e., $f_{2}/2\approx f_{1}$) versus the AC forcing voltage. As it can be seen the device is quickly driven to the nonlinear regime where the 2:1 internal resonance is triggered by the amplitude of $V_{AC}\geq$1 V and $\Omega_{r}$ =1.120448 MHz$\approx2f_{1}$. Therefore, signatures of nonlinear mode coupling due to the saturation phenomenon can be observed. This phenomenon leads to spill over of energy from the higher- frequency mode to the mode with the lower natural frequency. To obtain the numerical results, we have used the values of the system parameters in Table S1. The steady-state solution amplitude a1 in Equation (8) is used to achieve the simulation results illustrated in Figure S2. The figure demonstrates the modal amplitude a1 versus the nondimensionalized AC drive voltage $V_{AC}$ when $V_{DC}$=100000 and $\sigma_{2}=-\sigma_{1}$ ($\Omega_{1} =2\omega_{1}$). Upon using the real values for the system parameters, obtained from experimental measurements stated in Table S1, we can notice the activation of the nonlinear mode coupling by a response jump at $V_{AC}\geq$923.

Figure S3 shows the experimental and simulated frequency response of the first mode for three different drive voltages. The simulated amplitudes a1 are achieved using Equation (8). The observed coupling and associated nonlinear frequency response curves are the characteristics of the nonlinear systems with 2:1 internal resonance. Despite the actuation of the system at the excitation frequency in the vicinity of the spring-mode resonant frequency, the pendulum mode responds at half the excitation frequency. As the electrostatic voltage is increased, the microresonator starts to experience more nonlinear mode coupling, where the vibrational amplitude splits and two peaks of the vibrational deflection emerge in the vicinity of the pendulum-mode resonant frequency. The splitting of the resonance curve is a consequence of the nonlinear mode coupling due to the 2:1 internal resonance.

| Experiment  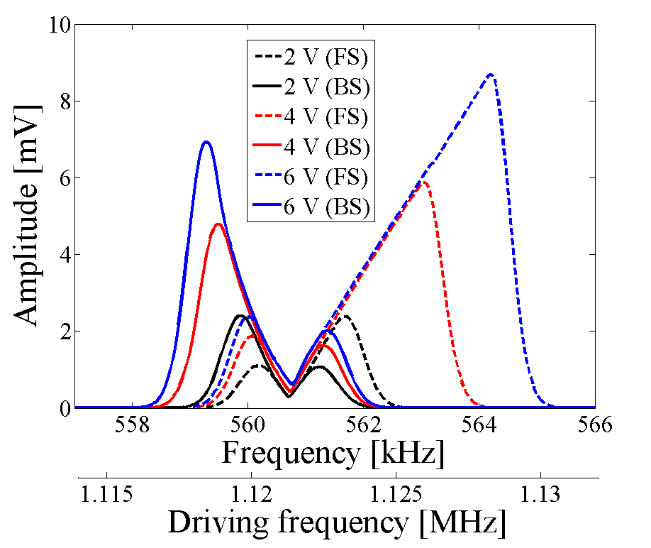  (a) | Simulation  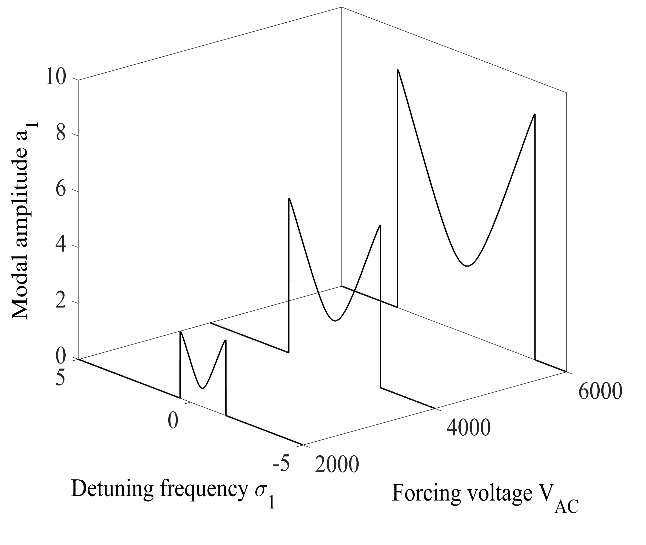  (b) |
| --- | --- |

Figure S3. The measured nonlinear frequency transmission responses for the H-shaped microresonator. (a) Experimental measurements; and (b) Simulation of the amplitude $a_{1}$ versus the detuning forcing parameter $\sigma_{1}$.


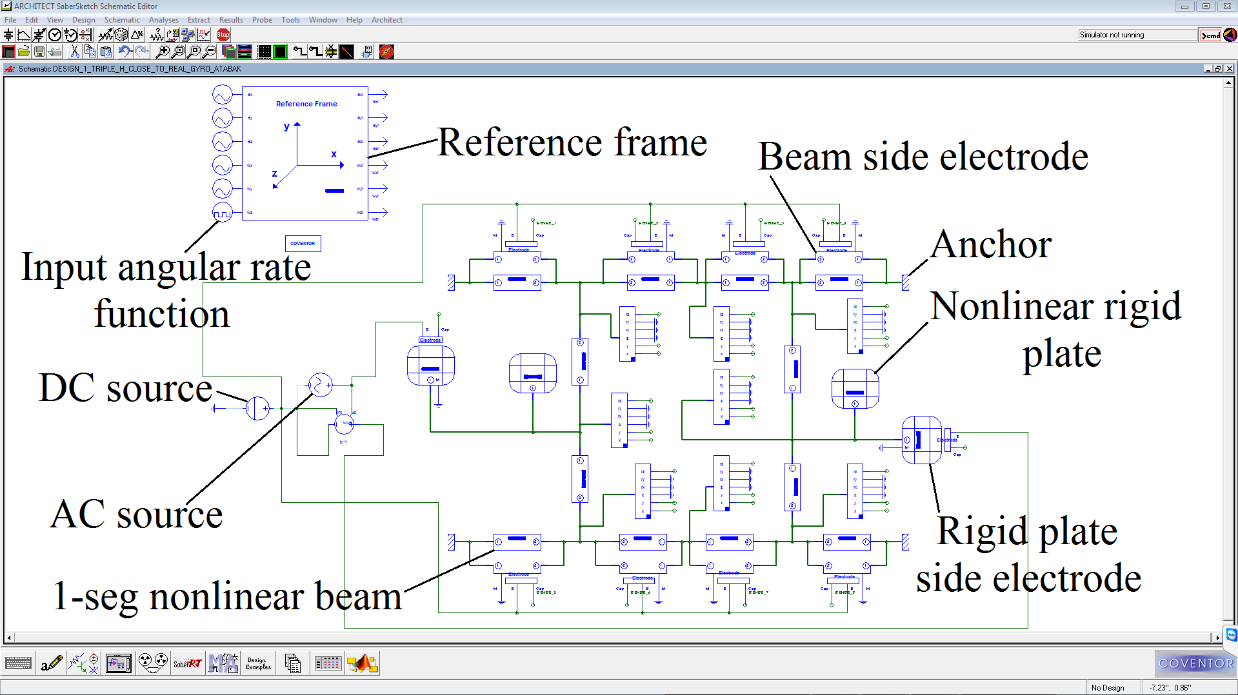


Figure S4. Complete system schematic of the microresonator in CoventorWare Architect.

## Numerical simulation results

The device behavior was numerically simulated in CoventorWare Architect. Reduced-order simulations run in significantly shorter times (more than 10× faster) than similar simulations based on finite element models, which in many cases even fail to converge. The Architect module in CoventorWare allows for direct, nonlinear reduced-order modelling of structures. A high-level schematic of the detailed structural design in Architect environment is shown in Figure S4.

The presence of the saturation phenomenon, and consequently quadratic couplings, in the microresonator dynamics can be confirmed in Figure S5. The figure illustrates the X-(spring) and Y-(pendulum) modal amplitudes as the AC voltage is changed from 0-40 V while the forcing frequency is secured at 1.052 MHz. It reveals that the linear changes in the spring-mode amplitude (X) stop at $V_{AC}=10 V$. Instead the pendulum-mode response (Y) jumps from zero-static equilibrium and grows further by an increase in the actuation levels. The time-domain responses associated with the region A in Figure S5(a), i.e., for the case of $V_{AC}=30 V$, are demonstrated in Figure S5(b). It implies the energy transfer from the spring-mode (X) to the pendulum-mode (Y) after 1.75 msec.

| 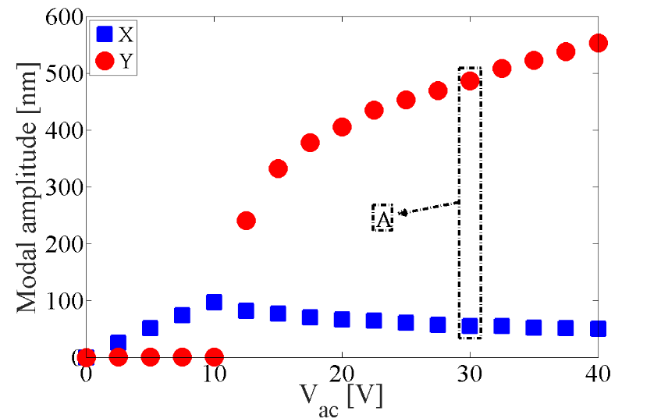 | 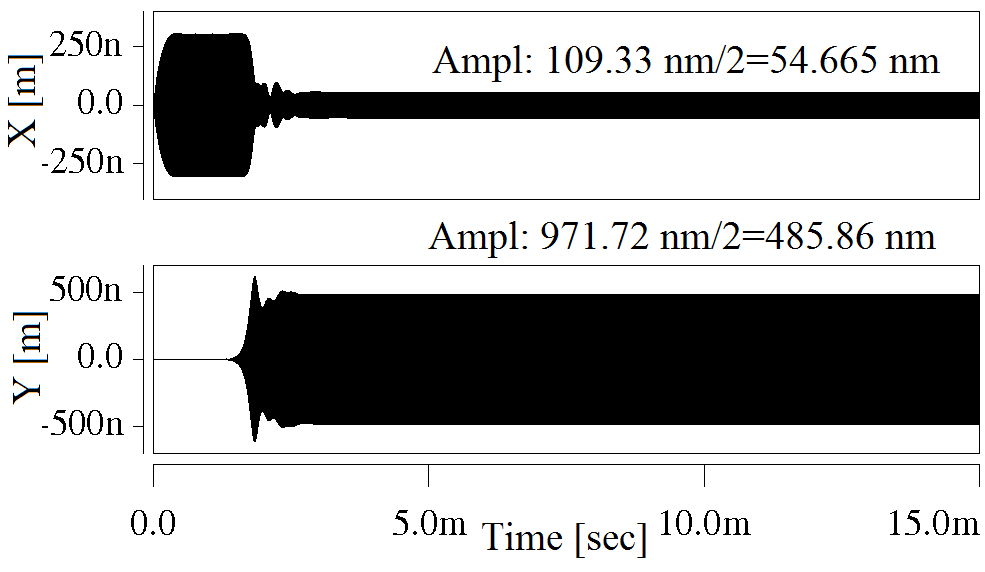 |
| --- | --- |
| (a) | (b) |

Figure S5. Transient simulation results for the H-shaped microresonator, where $V_{DC}=70 V$ and ${}_{exc}=1.052 MHz$. (a) the steady-state modal amplitudes of X and Y extracted from repeated transient simulations for $V_{AC}=[0 40] V$. (b) the oscillation amplitudes of the spring-mode (X) and the pendulum-mode (Y) for region A corresponding to $V_{AC}=30 V$.

| 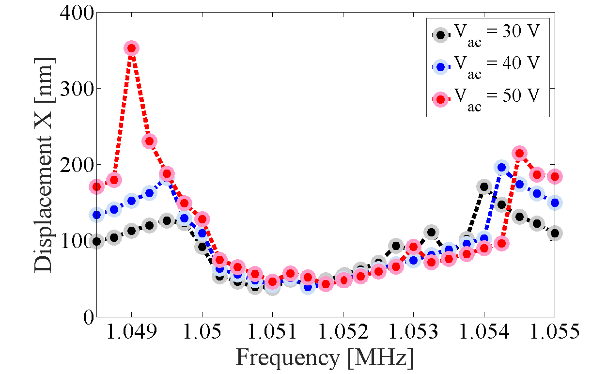  (a) | 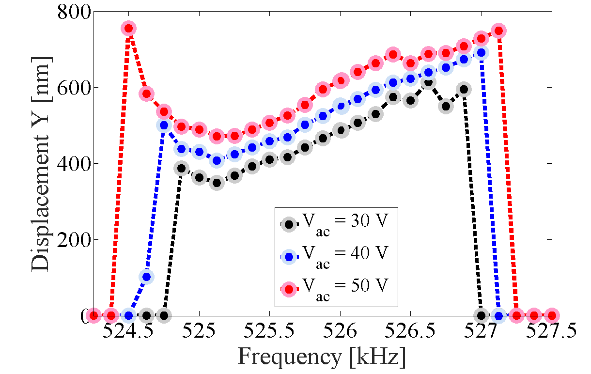  (b) |
| --- | --- |

Figure S6. Simulated nonlinear frequency response curves for the H-shaped microresonator achieved via Architect (a) the spring mode and (b) the pendulum mode.

Figure S6 shows the nonlinear frequency response plots for various AC levels $V_{AC}=30, 40$ and $50 V$. This figure is created from measuring the steady-state amplitudes of the signals (X and Y) for different excitation frequencies. It should be mentioned that the excitation frequency is changed in the vicinity of the spring mode resonant frequency (f_2_=1.0548 MHz). However, the vibrational modes respond differently. The spring-mode in Figure S6(a) resonates at the frequency of the excitation, although the pendulum-mode amplitudes shown in Figure S6(b) are captured at half the excitation frequency.

Figure S7 exhibits the dynamical behavior of the microresonator as it is opposed to the input rate of $\Omega_{z}$= 1.5 RPS (86 DPS), when $V_{AC}=20 V$. The assigned rate profile can be seen in the figure, which is constructed by 20-msec delay, a ramp function with constant angular acceleration 1910 deg/sec^2^). As it can be observed, the microresonator reacts to the input rate with an increase in both spring- and pendulum response amplitudes.


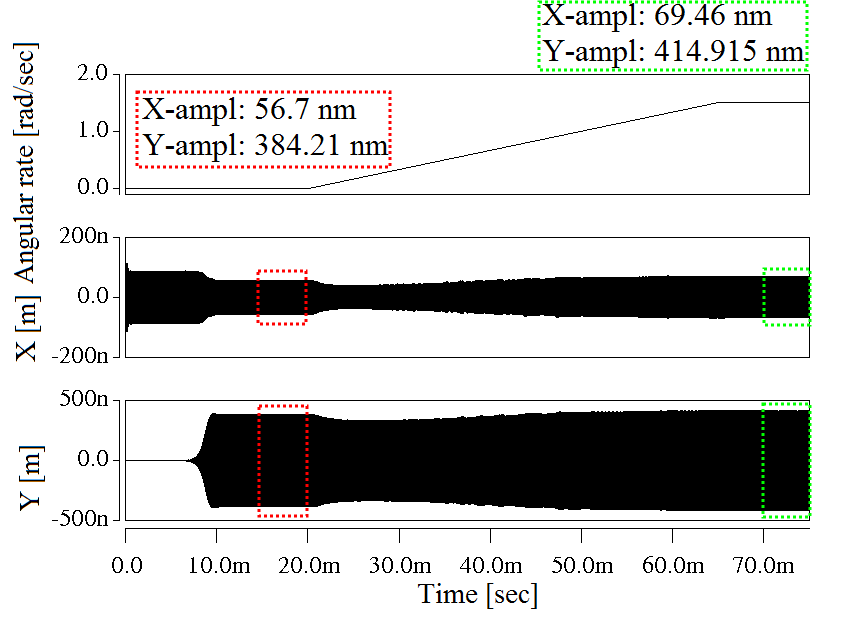


Figure S7. Numerical simulation for the time-domain response of the microresonator, when the applied angular velocity ${}_{Z}$ equals to 1.5 RPS (or 86 DPS).
